# Clinical efficacy of combined orthodontic and implant-supported prosthodontic treatment for dentition defects with dentofacial deformities

**DOI:** 10.1097/MD.0000000000047506

**Published:** 2026-01-30

**Authors:** Jian Yang, Haiying Sheng, Rongzhi Wei

**Affiliations:** a Department of Stomatology, Xinhua Hospital, Beijing City, China.

**Keywords:** dentition defects, dentofacial deformities, implant-supported prosthodontic restoration, orthodontic pretreatment, patient satisfaction, peri-implant health

## Abstract

This study aims to evaluate the clinical outcomes associated with orthodontic treatment combined with implant-supported prosthodontic restoration in patients with dentition defects accompanied by dentofacial deformities. This retrospective study included 104 patients treated between September 2019 and September 2024. Patients were assigned to a control group (n = 53), who received implant-supported prosthodontic restoration alone, or an observation group (n = 51), who underwent orthodontic treatment prior to implant placement. Baseline characteristics, including age, sex, bone density, and periodontal health, were comparable between groups. Clinical outcomes were assessed after 12 months of follow-up, including masticatory function, occlusal relationship, aesthetic satisfaction, peri-implant soft tissue health, implant survival, postoperative complications, and patient-reported satisfaction. At 12 months, the observation group showed significantly higher masticatory function and aesthetic satisfaction scores compared with the control group. A greater proportion of patients in the observation group achieved balanced and stable occlusal relationships. Peri-implant soft tissue conditions were more favorable, with shallower probing depths and a higher proportion of sites maintaining probing depth ≤ 4 mm. Implant survival rates were high in both groups, with a slightly higher rate observed in the observation group. Postoperative complications, including mucositis, swelling, and prosthesis fracture, occurred less frequently in patients who received orthodontic pretreatment. Patient-reported overall satisfaction, assessed using a 5-point Likert scale, was also significantly higher in the observation group. Within the limitations of this retrospective study, the findings suggest that orthodontic treatment performed prior to implant-supported prosthodontic rehabilitation is associated with improved functional outcomes, more favorable peri-implant soft tissue conditions, and higher patient satisfaction in patients with dentition defects and dentofacial deformities. This combined approach may be a beneficial strategy for comprehensive oral rehabilitation in complex clinical cases.

## 1. Introduction

Dentition defects accompanied by dentofacial deformities represent a complex clinical condition that significantly compromises oral function, facial aesthetics, and psychological well-being. These defects often result from congenital anomalies, trauma, periodontal disease, or tooth loss secondary to caries and malocclusion. They can lead to masticatory inefficiency, occlusal instability, altered facial symmetry, and progressive alveolar bone resorption, ultimately affecting the quality of life and social confidence of affected individuals. Conventional prosthetic approaches, while capable of restoring missing teeth, frequently fail to address the underlying occlusal disharmony and skeletal imbalance characteristic of dentofacial deformities, thereby limiting long-term stability and functional outcomes.^[1,2]^ Implant-supported prosthodontic rehabilitation has become the gold standard for the restoration of partial or complete edentulism due to its superior biomechanical stability, preservation of alveolar bone, and high long-term survival rates compared with traditional fixed or removable prostheses. However, successful implant restoration requires precise implant positioning, adequate bone volume, and harmonious occlusion. In patients with concomitant malocclusion or dentofacial asymmetry, the absence of optimal interarch relationships often leads to non-axial implant loading, peri-implant soft tissue tension, and aesthetic compromise.^[3,4]^ These unfavorable conditions may predispose patients to complications such as peri-implant mucositis, bone resorption, and prosthesis failure, thus underscoring the need for a multidisciplinary treatment strategy that integrates orthodontic correction with implant-based rehabilitation.

Orthodontic intervention prior to implant placement aims to redistribute interdental spaces, correct tooth inclination, and establish a functional occlusal plane, thereby facilitating prosthetically guided implant positioning. By realigning the adjacent dentition and optimizing intermaxillary relationships, orthodontic treatment improves load distribution during mastication and enhances the esthetic and biomechanical integration of implant-supported restorations. Recent studies have demonstrated that orthodontically assisted implant site development can enhance both hard and soft tissue morphology, reducing the necessity for bone grafting and improving peri-implant tissue health. Furthermore, interdisciplinary planning between orthodontists, implant surgeons, and prosthodontists has been shown to reduce technical complications, improve esthetic outcomes, and increase patient satisfaction in complex restorative cases.^[5,6]^ Despite growing evidence supporting the integration of orthodontic and implant-based therapies, clinical studies focusing specifically on patients with dentition defects accompanied by dentofacial deformities remain limited. Most existing research has concentrated on isolated orthodontic correction or implant rehabilitation, rather than assessing their combined impact on occlusal function, peri-implant soft tissue stability, and patient-centered outcomes. Moreover, there is a paucity of data directly comparing the efficacy and complication profile between conventional implant restoration and orthodontic-assisted implant-supported prosthodontic treatment in such patients.^[7–9]^

The present study was therefore designed to evaluate the clinical efficacy and patient-centered benefits of combining orthodontic treatment with implant-supported prosthodontic rehabilitation in individuals with dentition defects and dentofacial deformities. By comprehensively analyzing functional outcomes, implant stability, peri-implant tissue health, postoperative complications, and patient satisfaction, this study aims to provide evidence for a more integrated and personalized approach to oral rehabilitation, ultimately promoting improved long-term functional and esthetic outcomes.

## 2. Methods

### 2.1. Study design

This study was approved by the Ethics Committee of Xinhua Hospital. The present retrospective study enrolled patients diagnosed with dentition defects accompanied by dentofacial deformities who received treatment at our institution between September 2019 and September 2024 (No. 2024-XHH-12). Written informed consent was obtained from all participants. According to the treatment modality, patients were divided into 2 groups: the control group underwent conventional implant-supported prosthetic restoration, whereas the observation group received orthodontic treatment prior to implant placement. Approximately 6 months after orthodontic therapy, implant prosthetic restoration was performed following the same procedures as those used in the control group, depending on the patient’s dental defect condition. Inclusion criteria were as follows: a confirmed diagnosis of dentition defects with dentofacial deformities based on clinical and radiographic examinations; absence of systemic diseases or oral conditions contraindicating implant surgery; good oral hygiene and compliance with follow-up requirements; and complete clinical and imaging data. Exclusion criteria included: presence of active periodontal disease or oral infection; severe systemic disorders such as uncontrolled diabetes or cardiovascular disease; history of maxillofacial trauma or surgery affecting occlusal function; and pregnancy or lactation during treatment. The study was conducted in accordance with the ethical standards outlined in the Declaration of Helsinki and was approved by the Institutional Medical Ethics Committee. Informed consent was obtained from all participants.

### 2.2. Treatment protocol

After hospital admission, all patients underwent comprehensive clinical and radiographic evaluations, including assessments of residual dentition, dental arch morphology, and occlusal relationships. Panoramic radiography and cone-beam computed tomography were performed to evaluate alveolar bone density, height, and morphology at potential implant sites. Based on the diagnostic findings, an individualized treatment plan and prosthetic restoration model were formulated for each patient.

Control group (implant-supported prosthetic restoration alone): Patients in the control group received conventional implant-supported prosthetic restoration. Preoperatively, detailed imaging examinations, including cone-beam computed tomography and periapical radiographs, were performed to assess periodontal status and alveolar bone conditions. Following local anesthesia and routine disinfection, an incision was made at the predetermined implant site to fully expose the alveolar bone. Guided drilling was performed according to the preoperative plan, with incremental enlargement of the osteotomy to the desired depth and diameter under continuous saline irrigation to prevent thermal injury. Implants were then inserted at the designated torque, and healing abutments or cover screws were placed according to gingival thickness and implant depth. The surgical site was sutured, and postoperative instructions were provided.

Observation group (orthodontic treatment followed by implant-supported prosthetic restoration): In addition to the procedures described for the control group, patients in the observation group underwent orthodontic treatment before implant placement. Fixed orthodontic appliances, such as straight-wire or edgewise archwire systems, were applied to redistribute interdental spaces, correct malocclusion, and adjust axial tooth inclination. Extruded teeth were repositioned by controlled intrusion, and occlusal alignment was refined. The orthodontic phase lasted approximately 6 months, after which implant placement and prosthetic restoration were performed using the same standardized surgical protocol as in the control group.

All implant surgeries were performed by the same surgical team comprising experienced implantologists and operating nurses to ensure procedural consistency. Orthodontic treatments were conducted by the same orthodontic specialists and nursing staff. The prosthetic restorations in both groups were fabricated using cobalt–chromium alloy frameworks to maintain material uniformity and biomechanical stability. Throughout the treatment course, postoperative care included antibiotic prophylaxis, oral hygiene instruction, and periodic clinical and radiographic follow-up to monitor osseointegration, prosthesis fit, and occlusal function. Any postoperative complications, such as mucositis, implant loosening, or prosthetic fracture, were recorded and managed according to established clinical guidelines.

### 2.3. Data collection

Data were retrospectively collected from the medical records of patients diagnosed with dentition defects accompanied by dentofacial deformities who received implant-supported prosthetic restoration at our institution between September 2019 and September 2024. Two independent investigators, blinded to the group allocation, extracted all relevant data using standardized forms. Baseline data included age, sex, disease duration, alveolar bone height, bone density (Hounsfield units, HU), smoking history, periodontal health, and systemic comorbidities (hypertension or diabetes), which were used to evaluate the comparability of the 2 groups. Functional and aesthetic outcomes were assessed 12 months after prosthetic restoration, including masticatory function (0–10 score), occlusal relationship (balanced contact), and aesthetic satisfaction evaluated using a 10-point visual analog scale. Implant stability and osseointegration were evaluated using implant survival rate, probing depth (PD) at 6 sites per implant (PD ≤ 4 mm considered healthy), and the proportion of sites with shallow pockets. Postoperative complications such as pain, swelling, mucositis, implant loosening, and prosthesis fracture were recorded during the 12-month follow-up. Patient-reported outcomes included an assessment of overall satisfaction at 12 months, which was evaluated using a 5-point Likert scale. All data were verified for completeness and consistency before statistical analysis.

### 2.4. Statistical analysis

All data were analyzed using SPSS version 28.0 (IBM Corp., Armonk). Continuous variables were tested for normal distribution using the Shapiro–Wilk test and expressed as mean ± standard deviation. Comparisons between the 2 groups were performed using the independent-samples t-test for normally distributed data and the Mann–Whitney *U* test for non-normal data. Categorical variables were expressed as counts and percentages (n [%]) and compared using the χ^2^ test or Fisher exact test, as appropriate. All statistical tests were 2-tailed. A *P*-value <.05 was considered statistically significant.

Sample size estimation was not performed a priori because this was a retrospective study, and the sample size was determined by the number of eligible patients treated during the predefined study period. To address statistical validity, a post hoc power analysis was conducted based on the primary outcome measure (masticatory function score). With the observed effect size and a 2-sided significance level of 0.05, the achieved sample size provided adequate statistical power (>80%) to detect a clinically meaningful difference between the 2 groups.

## 3. Results

### 3.1. Baseline characteristics

A total of 104 patients diagnosed with dentition defects accompanied by dentofacial deformities were included in the study, comprising 53 patients in the control group and 51 patients in the observation group. The baseline demographic and clinical characteristics of both groups are presented in Table 1. There were no statistically significant differences between the 2 groups in terms of age, sex distribution, disease duration, alveolar bone height, bone density, smoking history, periodontal health, or systemic comorbidities (*P* >.05), demonstrating good baseline comparability. The mean age of the control group was 39.5 ± 8.8 years, and that of the observation group was 39.9 ± 8.4 years (*P* = .813). The male-to-female ratio was 26:27 in the control group and 24:27 in the observation group (*P* = .839). The mean disease duration was 3.8 ± 1.6 years in the control group and 3.7 ± 1.4 years in the observation group (*P* = .736). Radiographic evaluation revealed similar alveolar bone height between the 2 groups (11.36 ± 1.72 mm vs 11.41 ± 1.65 mm, *P* = .880). The mean bone density, measured in HU, was 841.3 ± 97.6 HU in the control group and 854.9 ± 103.5 HU in the observation group (t = 0.690, *P* = .492). In addition, the prevalence of smoking history was 15.1% in the control group and 13.7% in the observation group (*P* = .843). The distribution of periodontal health status (good/moderate/poor) and systemic diseases (hypertension or diabetes) also showed no significant intergroup differences (*P* = .918 and *P* = .802, respectively).

**Table 1:** Baseline demographic and clinical characteristics of the 2 groups (Mean ± SD or n (%)).

Parameter	Control group (n = 53)	Observation Group (n = 51)	t/ χ^2^ value	*P*-value
Age (year)	39.5 ± 8.8	39.9 ± 8.4	0.237	.813
Sex (male/female)	26/ 27	24/ 27	0.042	.839
Disease duration (year)	3.8 ± 1.6	3.7 ± 1.4	0.339	.736
Alveolar bone height (mm)	11.36 ± 1.72	11.41 ± 1.65	0.151	.880
Bone density (HU)	841.3 ± 97.6	854.9 ± 103.5	0.690	.492
Smoking history, n (%)	8 (15.1%)	7 (13.7%)	0.039	.843
Periodontal health (good/moderate/poor)	32/ 17/ 4	31/ 15/ 5	0.172	.918
Systemic disease (hypertension, diabetes), n (%)	6 (11.3%)	5 (9.8%)	0.063	.802

HU = Hounsfield units, SD = standard deviation.

### 3.2. Functional and aesthetic outcomes

Both groups showed significant postoperative improvements in masticatory function, occlusal relationship, and aesthetic satisfaction; however, patients in the observation group, who underwent orthodontic treatment before implant-supported prosthetic restoration, demonstrated superior outcomes in all parameters. The mean masticatory function score increased markedly after treatment in both groups. At 12 months, the observation group achieved a mean score of 9.15 ± 0.71, which was significantly higher than that of the control group (8.27 ± 0.82; *P* <.001). In terms of occlusal relationship, 92.2% of patients in the observation group achieved balanced occlusal contact and stable intercuspal position, compared with 77.4% in the control group (*P* = .037). Regarding aesthetic satisfaction, the observation group reported a higher mean visual analog scale score (9.31 ± 0.64) compared with the control group (8.38 ± 0.76; *P* <.001). Patients who received orthodontic correction before implant placement exhibited improved dental alignment, occlusal balance, and facial aesthetics (Table 2).

**Table 2:** Comparison of functional and aesthetic outcomes between groups (Mean ± SD).

Parameter	Control Group (n = 53)	Observation Group (n = 51)	t/ χ^2^ value	*P*-value
Masticatory function score (0–10)	8.27 ± 0.82	9.15 ± 0.71	5.841	<.001
Occlusal relationship-balanced contact, n (%)	41 (77.4%)	47 (92.2%)	4.372	.037
Aesthetic satisfaction (VAS, 0–10)	8.38 ± 0.76	9.31 ± 0.64	6.737	<.001

SD = standard deviation, VAS = visual analog scale.

### 3.3. Implant stability and osseointegration

All implants in both groups demonstrated good osseointegration and functional stability. However, the observation group, which received orthodontic treatment prior to implant-supported prosthetic restoration, exhibited superior outcomes in terms of peri-implant soft tissue health and implant survival (Table 3). The implant survival rate, defined as the absence of implant mobility, pain, peri-implant infection, or radiographic bone resorption exceeding one-third of the implant length, was 100.0% (51/51) in the observation group and 96.2% (51/53) in the control group. Although no statistically significant difference was detected (*P* = .152), the observation group showed a slightly higher survival trend and fewer early complications such as mucositis or marginal bone loss. The mean PD around implants was 3.12 ± 0.49 mm in the observation group and 3.46 ± 0.55 mm in the control group, with the difference reaching statistical significance (t = 3.324, *P* = .001). Notably, 92.2% of implant sites in the observation group maintained PD ≤ 4 mm, compared with 81.1% in the control group.

**Table 3:** Comparison of implant stability and osseointegration between groups (Mean ± SD or n [%]).

Parameter	Control group (n = 53)	Observation group (n = 51)	t/ χ^2^ value	*P*-value
Implant survival rate, n (%)	51 (96.2%)	51 (100.0%)	1.962	.161
Probing depth (mm)	3.46 ± 0.55	3.12 ± 0.49	3.324	.001
Sites with PD ≤ 4 mm, n (%)	43 (81.1%)	47 (92.2%)	2.712	.100

SD = standard deviation.

### 3.4. Complication results

During the 12-month postoperative follow-up, all patients were carefully monitored for local and systemic complications, including pain, swelling, mucositis, implant loosening, and prosthesis fracture. As shown in Table 4, the overall incidence of postoperative complications was low in both groups, and all events were mild to moderate in severity and resolved after symptomatic management without requiring additional surgery. The total complication rate in the control group was 22.6% (12/53), significantly higher than 7.8% (4/51) observed in the observation group (χ^2^ = 4.372, *P* = .037). Among the specific events, transient pain occurred in 4 patients (7.5%) in the control group and 2 patients (3.9%) in the observation group (*P* = .428). Swelling was reported in 4 patients (7.5%) in the control group and 1 patient (2.0%) in the observation group (*P* = .183). Mucositis developed in 2 cases (3.8%) in the control group and 1 case (2.0%) in the observation group (*P* = .581). Implant loosening and prosthesis fracture were rare, each observed in 1 patient (1.9%) in the control group and none in the observation group (*P* = .324 for both comparisons). No cases of peri-implant infection, radiographic bone resorption beyond one-third of the implant length, or systemic adverse events were detected in either group.

**Table 4:** Postoperative complications (n, %).

Complication Type	Control group (n = 53)	Observation group (n = 51)	χ^2^ value	*P*-value
Pain	4 (7.5%)	2 (3.9%)	0.628	.428
Swelling	4 (7.5%)	1 (2.0%)	1.772	.183
Mucositis	2 (3.8%)	1 (2.0%)	0.305	.581
Implant loosening	1 (1.9%)	0 (0.0%)	0.972	.324
Prosthesis Fracture	1 (1.9%)	0 (0.0%)	0.972	.324
Total complication rate	12 (22.6%)	4 (7.8%)	4.372	.037

### 3.5. Overall satisfaction

At 12 months after restoration, overall treatment satisfaction was significantly higher in the observation group compared with the control group. As shown in Table 5, the mean satisfaction score, measured using a 5-point Likert scale, was 4.4 ± 0.5 in the observation group and 3.8 ± 0.6 in the control group (t = 5.529, *P* <.001). Furthermore, the proportion of patients who reported being “satisfied” or “very satisfied” with their treatment outcomes – including functional, aesthetic, and psychological aspects – was significantly higher in the observation group (90.2% [46/51]) than in the control group (75.5% [40/53]) (χ^2^ = 3.937, *P* = .047).

**Table 5:** Comparison of overall satisfaction between groups.

Parameter	Control group (n = 53)	Observation group (n = 51)	t/ χ^2^ value	*P*-value
Overall satisfaction score (1–5 Likert)	3.8 ± 0.6	4.4 ± 0.5	−5.529	.000
Satisfied/ very satisfied, n (%)	40 (75.5%)	46 (90.2%)	3.937	.047

SD = standard deviation.

## 4. Discussion

This study evaluated the clinical efficacy of an integrated approach that combines preimplant orthodontic treatment with implant-supported prosthodontic rehabilitation in patients with dentition defects accompanied by dentofacial deformities. The principal findings were as follows. First, despite comparable baseline characteristics between the 2 groups, the combined orthodontic–implant strategy resulted in superior postoperative functional and aesthetic outcomes, including higher masticatory function scores, more stable occlusion, and higher aesthetic satisfaction at 12 months. Second, peri-implant soft tissue conditions were more favorable in the combined-treatment group, as reflected by significantly shallower PD and a higher proportion of sites with PD ≤ 4 mm. Third, although implant survival was high in both groups, there was a trend toward higher survival and fewer peri-implant complications in the group receiving orthodontic preparation. Fourth, the total complication rate over 12 months was significantly lower in the combined-treatment group. Finally, patient-reported satisfaction scores were significantly higher in the combined-treatment group, both in terms of mean Likert scale score and the proportion of patients reporting themselves as satisfied or very satisfied.

The superiority of functional outcomes in the observation group can be interpreted in biomechanical and occlusal terms. Malalignment and improper interproximal spacing in partially edentulous arches can lead to nonideal implant positioning and suboptimal occlusal load distribution, which in turn may compromise both chewing efficiency and long-term stability of the restoration. Preimplant orthodontic correction redistributes interdental spaces, repositions extruded or tilted adjacent teeth, and establishes a more physiologic occlusal scheme. This facilitates prosthetically driven implant placement and angulation within zones of favorable alveolar height and bone density, ultimately enabling more stable intercuspal contact and improved masticatory function. Similar concepts have been emphasized in recent interdisciplinary orthodontic–implant planning literature, which underscores that orthodontic space management and axial correction of neighboring teeth allow implants to be placed in prosthetically ideal positions, enhancing occlusal balance and functional loading after restoration.^[10]^

The present study also demonstrated a clinically relevant improvement in aesthetic satisfaction in the observation group. This likely reflects 2 mechanisms: orthodontic correction of dental crowding, rotation, or supra-eruption prior to implant placement improves tooth alignment and incisal edge position, which contributes to facial symmetry and lip support; and prosthesis emergence profile and soft tissue contours are easier to optimize when implant positioning is not constrained by malocclusion. Contemporary reports have highlighted that facial and dental esthetics in adult patients with dentofacial deformities often require multidisciplinary management rather than isolated prosthetic replacement, particularly because untreated malocclusion and midline asymmetry can undermine esthetic outcomes even when implant restorations are structurally sound.^[11]^ Importantly, the observation group also showed significantly better peri-implant soft tissue conditions, with a lower mean PD and a higher proportion of sites maintaining PD ≤ 4 mm at 12 months. Shallow peri-implant PD is generally interpreted as an indicator of favorable mucosal adaptation, reduced inflammation, and controlled biofilm accumulation. Maintenance of healthy peri-implant soft tissue is recognized as critical for long-term implant success, because mucosal thickness, keratinized mucosa width, and emergence profile geometry influence both esthetic stability and susceptibility to peri-implant mucositis and peri-implantitis.^[12,13]^ The current findings suggest that orthodontic preconditioning may indirectly promote healthier peri-implant soft tissues by enabling implants to be positioned within zones of adequate keratinized tissue and favorable bony support, and by reducing the need for compensatory prosthetic angulation that can create cleansability challenges. This interpretation is consistent with recent analyses indicating that implant site development, including orthodontically guided tooth movement or forced eruption, can enhance both hard tissue and soft tissue dimensions prior to implant placement, thereby supporting more stable soft tissue architecture around the final restoration.^[14,15]^

Although the implant survival rate did not differ significantly between the 2 groups over 12 months, it is notable that the combined-treatment group achieved 100.0% survival, compared with 96.2% in the control group, and also demonstrated fewer biologic and mechanical complications. Peri-implant mucositis, implant loosening, and prosthesis fracture were numerically less frequent in patients who received orthodontic preparation. This observation aligns with the concept that strategic implant positioning and favorable occlusal contact reduce non-axial loading during function, which in turn decreases micromovements and mechanical strain on both fixtures and superstructures. Prior retrospective work has indicated that prosthesis design, emergence profile, and implant positioning are associated with the development of peri-implant pathology, whereas malposition alone is not necessarily the dominant risk factor when other factors are controlled.^[16]^ The present results extend that discussion by suggesting that orthodontic preparation may reduce the need for compromised implant angulation or overcontoured prosthetic emergence profiles, both of which have been associated with plaque retention and peri-implant soft tissue inflammation.^[12]^

Another relevant finding of this study is the significantly lower overall complication rate in the observation group. The reduction in pain, swelling, and soft tissue inflammation, although not individually statistically significant for each event, was collectively reflected in a significantly lower total complication rate. This pattern further supports the view that preimplant orthodontic alignment may decrease the biologic stress imposed on peri-implant tissues during healing and functional loading. Multidisciplinary strategies that coordinate orthodontics, implant surgery, and prosthodontics have recently been advocated for patients with complex dentofacial conditions, particularly in cases where skeletal or occlusal discrepancies increase soft tissue tension or create traumatic contacts after restoration.^[17]^ Finally, patient-reported outcomes demonstrated a clear advantage for the combined-treatment protocol. At 12 months, patients in the observation group reported higher overall satisfaction scores and a higher proportion of “satisfied” or “very satisfied” ratings. The improvement in subjective satisfaction is clinically meaningful, because perceived comfort, esthetics, and social confidence are major determinants of treatment acceptance and long-term adherence to maintenance regimens. Recent work has emphasized that, particularly in adult patients with malocclusion and dentofacial asymmetry, interdisciplinary rehabilitation that addresses both occlusal stability and facial harmony is associated with improved psychosocial outcomes and quality of life.^[18,19]^ Taken together, the current data indicate that preimplant orthodontic correction does not simply optimize mechanical parameters; it also confers measurable benefits in patient-reported quality of life.

Comparison with previously published studies over the past 3 years further supports the present findings. Interdisciplinary treatment models have been described as increasingly necessary in adult patients who present with partial edentulism, occlusal instability, and dentofacial disharmony, reflecting a demographic shift in which adult orthodontic demand continues to rise. Schneider et al (2024) emphasized that complex adult cases often require coordinated orthodontic, implant, and restorative planning to achieve long-term stability and acceptable esthetics, because isolated prosthetic replacement without correction of malocclusion may perpetuate functional overload and esthetic asymmetry.^[1]^ From a biologic standpoint, current literature has underscored the importance of peri-implant soft tissue phenotype – keratinized mucosa width, mucosal thickness, and supracrestal tissue height – for both esthetic stability and long-term peri-implant health. Monje et al (2023) reported that adequate peri-implant mucosal dimensions are fundamental for maintaining soft tissue integrity and esthetic predictability, and that deficiencies often require soft tissue augmentation or other site development measures prior to final restoration.^[12]^ Orthodontically assisted site development, including orthodontic forced eruption, has been proposed as a means to vertically augment hard and soft tissue before implant placement, thereby creating more favorable ridge morphology and emergence profile. Ardakani et al (2024) concluded that orthodontic forced eruption can enhance both hard and soft tissue at future implant sites, facilitating esthetic implant placement and reducing the need for extensive grafting in selected cases.^[14]^ The present study did not specifically evaluate forced eruption maneuvers, but the observed reduction in PD and peri-implant soft tissue inflammation in the combined-treatment group is consistent with the idea that orthodontically optimized sites can support healthier peri-implant tissue conditions.

Furthermore, Liaw et al (2024) described that interdisciplinary orthodontic–implant planning, including the use of skeletal anchorage to control tooth movement, improves control over implant positioning in patients with malocclusion and partial edentulism, and reduces unfavorable loading patterns after prosthetic rehabilitation.^[10]^ This aligns with the lower overall complication rate and trend toward higher implant survival in the observation group of the present study. In parallel, recent systematic analyses indicate that orthodontic involvement in complex prosthetic or maxillofacial rehabilitation improves prosthesis retention, occlusal stability, and patient comfort, ultimately contributing to higher self-reported satisfaction and quality of life.^[19]^ Collectively, contemporary findings from the past 3 years support the conclusion that coordinated orthodontic and implant-supported prosthodontic treatment offers functional, biologic, and psychosocial advantages over implant rehabilitation alone in patients with dentition defects and dentofacial deformities.

The clinical implications of these findings are direct. In patients with dentition defects and concomitant dentofacial deformity, implant restoration should not be approached as an isolated replacement of missing teeth. Instead, preimplant orthodontic alignment should be considered when there is crowding, tipping, supra-eruption, or malocclusion that would compromise implant positioning or load distribution. Incorporating orthodontic correction prior to implant placement may improve masticatory function, reduce peri-implant soft tissue stress, lower complication rates, and increase patient satisfaction at 12 months, suggesting that this protocol should be incorporated into routine treatment planning for complex restorative cases. This study has several strengths. First, both groups were comparable at baseline with respect to demographic and clinical variables, including bone quality and periodontal health, which reduces selection bias and supports the internal validity of between-group comparisons. Second, outcomes were evaluated across multiple clinically relevant domains – objective function, peri-implant biology, complication profile, and patient-reported satisfaction – rather than relying solely on implant survival. Third, follow-up extended to 12 months after final restoration, a time frame that captures early mechanical and biologic adaptation to functional loading and provides initial insight into peri-implant tissue stability.

The study also has limitations. The retrospective design inevitably introduces the possibility of selection bias. Treatment allocation was not randomized but based on clinical decision-making and patient preference, which may have influenced group composition despite comparable baseline characteristics. Second, information bias cannot be entirely excluded, as data were collected from existing medical records, and variations in documentation accuracy or completeness may have affected outcome assessment. Third, although major baseline variables were comparable between groups, residual confounding factors – such as patient compliance with oral hygiene instructions, occlusal force distribution, parafunctional habits, and long-term maintenance behaviors – could not be fully controlled and may have influenced clinical outcomes.

## 5. Conclusions

This study suggests that combining orthodontic treatment with implant-supported prosthodontic restoration is associated with improved clinical outcomes in individuals with dentition defects and dentofacial deformities. Compared with implant restoration alone, the combined approach resulted in superior masticatory efficiency, better occlusal balance, higher aesthetic satisfaction, healthier peri-implant soft tissues, fewer postoperative complications, and greater overall patient satisfaction. These findings support the integration of orthodontic correction into preimplant treatment planning to optimize both functional and aesthetic rehabilitation outcomes in complex restorative cases.

## Author contributions

**Conceptualization:** Jian Yang, Haiying Sheng, Rongzhi Wei.

**Data curation:** Jian Yang, Haiying Sheng, Rongzhi Wei.

**Formal analysis:** Jian Yang, Haiying Sheng, Rongzhi Wei.

**Funding acquisition:** Jian Yang, Haiying Sheng, Rongzhi Wei.

**Investigation:** Rongzhi Wei.

**Writing – original draft:** Jian Yang, Haiying Sheng, Rongzhi Wei.

**Writing – review & editing:** Jian Yang, Haiying Sheng, Rongzhi Wei.
